# Recruitment of rural healthcare professionals for live continuing education

**DOI:** 10.3402/meo.v20.28958

**Published:** 2015-11-06

**Authors:** Ronnie Scott Holuby, Karen L Pellegrin, Anna Barbato, Anita Ciarleglio

**Affiliations:** 1 San Antonio Military Medical Center, San Antonio, TX, USA; 2 Daniel K. Inouye College of Pharmacy, University of Hawaii at Hilo, Hilo, HI, USA; 3 Oklahoma Heart Hospital, Oklahoma City, OK, USA

**Keywords:** marketing, continuing education, CE, geriatrics, rural health, medication safety, cost-effectiveness

## Abstract

**Introduction:**

The availability of rural healthcare is a growing concern in the United States as fewer healthcare providers choose to work in rural areas. Accessing quality continuing education (CE) for rural healthcare practitioners (HCPs) remains a challenge and may pose a barrier to quality care.

**Methods:**

To maximize attendance at a live, in-person, free CE program focusing on geriatric medication and issues specifically targeted to HCPs in rural areas, two methods were implemented sequentially. The first method used formal advertising implemented by a professional marketing service to promote CE events. The second method enlisted local healthcare organizations and physician groups to promote the CE event to their employees. Cost per attendee was calculated for comparison.

**Results:**

Professional marketing services recruited 31 HCPs (March 2011) and resulted in a per-participant recruitment cost of US$428.62. Local healthcare organizations and physician groups’ marketing recruited 48 HCPs (July–August 2011) and resulted in a per-participant recruitment cost of US$55.19.

**Discussion:**

Providing free CE coordinated through local healthcare organizations and physician groups was the most cost-effective method of recruiting rural HCPs for CE. Formal advertising added cost without increasing the number of participants per event. Although this is the first study of the cost-effectiveness of recruitment methods targeting HCPs in rural areas, results are consistent with research on cost-effectiveness of outreach to rural lay community members.

Continuing education (CE) for healthcare professionals is a licensure requirement for physicians, pharmacists, and many other healthcare providers. CE is essential to ensure that patients receive current and optimum care. Access to live CE is challenging for most healthcare practitioners (HCPs), particularly for those living and working in rural areas (1). Among the many barriers for rural HCPs are geographic isolation, cost, and time (2). In a survey of clinicians in rural areas, physicians reported that access to CE opportunities was only fair to good (i.e., 2.6 on a five-point scale where 1=poor, 2=fair, 3=good, 4=very good, and 5=excellent) (3).

Given limited access to CE, several studies have examined the impact of online or web-based programs in meeting the need for CE. Most HCPs (>90%) have computer access, despite the fact that rural HCPs are traditionally slow to adopt new technologies and continue to prefer face-to-face interactions (4, 5). Younger HCPs may be shifting toward web-based technology for participating in CE, and many prefer home study materials over traveling long distances for CE (5). Rural nurse practitioners in Canada expressed ‘distance’ as the most significant barrier to access to CE. They felt that networking face-to-face was also very important (6). HCPs in Kansas, including some in rural areas, rated interactive video just as effective as live presentations for CE (7). Canadian HCPs agreed that electronic CE can be effective if it is credible, applicable, and directed (8). Primary-care physicians were more likely than specialists to use the Internet for CE (9). The most important characteristic of online CE was quality of content, and the most disappointing aspect was lack of interaction (10).

Thus, although online CE offers convenience to rural HCPs, live CE that offers face-to-face interaction is also clearly valued. Given this, identification of optimal ways to reach rural HCPs to deliver such live CE is important to improving healthcare in rural areas.

## Medication safety in the elderly

It is widely understood that patients over the age of 65 take more medication and experience more adverse reactions to medication than other age groups (11). Many elderly patients reside in rural areas, making healthcare critical because they constitute the largest group of healthcare consumers in the United States (11). Multiple programs and criteria have been developed to improve the care of geriatric patients and reduce the risks associated with inappropriate medication use. These criteria include Beers, STOPP, and START (12–14). Several studies demonstrate that even common medications (e.g., aspirin, warfarin, and metformin) cause adverse effects, increasing emergency room visits and hospitalization among elderly patients (15, 16).

Thus, a CE program was developed for rural HCPs to improve medication management in elderly patients. Supported by a US Department of Agriculture (USDA) grant to improve the health and safety of elderly residents in rural areas, this study compared the cost-effectiveness of two recruitment methods used to maximize attendance by rural HCPs at the live, in-person CE events on medication safety in the elderly.

## Methods

A 2-hour, live CE program was designed for physicians, pharmacists, and nurses to improve medication safety among elderly patients. The program included discussion of the physiologic changes with aging, inappropriate medication use in the geriatric population (based on Beers Criteria, STOPP, and START), medication monitoring (renal and hepatic), and adverse effect prevention. Patient education handouts were included with the program to assist HCPsin counseling their patients on safe medication use. All CE programs were free to attendees, and there was no obligation to participate. Two counties in Hawaii classified as ‘non-metro’ according to the USDA 2003 Rural-Urban Continuum Codes were targeted (i.e., Maui and Hawaii counties).

The live, in-person CE program was first promoted using a formal marketing service to maximize attendance. Four live events were held at various resort hotels as well as convenient, community-based places on Maui and Hawaii islands. Marketing experts designed promotional advertisements in the form of cards that were dropped off at community pharmacies and mailed to physicians who were located on these islands according to a professional directory. The cards contained a link to the website, where potential participants could get more information and register for the CE event.

The second phase of promotion used informal methods to target rural HCPs. Six live, in-person CE events were promoted through local healthcare organizations and physician group practices. No formal marketing was used for this phase. Healthcare organizations and physician groups that care for the rural population on Maui and Hawaii islands were contacted and agreed to internally promote the events, which were held at each respective organization at a time convenient for their clinicians.

## Results

Recruitment costs for the first phase (formal advertising) included fees paid to the marketing firm, development and maintenance of a CE website with detailed information on the upcoming events and registration instructions, costs associated with printing and mailing of promotional cards, and personnel costs associated with recruitment activities. The total recruitment cost was US$13,287. Over the course of the four events, there were 31 participants (i.e., eight participants per event). The per-participant cost was US$428.62

Recruitment costs for the second phase (informal recruitment through local healthcare providers) included personnel costs associated with recruitment activities, such as collaborating with healthcare provider groups to host CE events. The total recruitment cost was $2,649.11. Over the course of the six events, there were 48 participants (i.e., eight participants per event). The per-participant cost was $55.19.

## Discussion

The difficulty for rural HCPs to meet their CE needs is well documented (1, 2, 17, 18). Some HCPs give up rural practice because of challenges meeting CE requirements (19). Although online CE is a convenient option, many HCPs consider live, in-person CE to be important and valuable as a rural clinician. To promote and maximize attendance at CE programs on medication safety in the elderly, formal advertising from a professional marketing company was implemented, but it was not the most cost-effective method. Rather, informal promotion through local hospitals and physician groups was the more cost-effective method of recruiting rural HCPs to attend this CE program. These results are consistent with research on outreach to lay community members in rural areas, which also found that formal advertisement was the least cost-effective approach (20).

There are several potential limitations to these findings. Given that the college sponsoring the CE is relatively new (founded in 2006), it is possible that the lack of familiarity with the college among the target audience may have differentially affected the impact of the two recruitment methods. It is possible that the second phase of recruitment may have benefited from word of mouth generated by the first phase. Finally, season is a confounding variable as the formal advertisement method was implemented in late winter and early spring, whereas the informal method was implemented during summer months.

It is noteworthy that the average number of attendees per event was the same in both groups. Thus, the less-expensive, informal promotion method did not increase the cost of delivering the CE program itself through necessitation of more events to achieve the same attendance. The informal promotional approach through local healthcare providers was likely successful for at least two reasons. First, the informal approach optimized convenience for the providers. The CE events were scheduled on a day, time, and place optimal for their clinicians. In all cases, the local provider requested that the event be held on-site. This choice of location made it very convenient for the clinicians to attend. Second, the informal method involved a more personal interaction with the provider that likely inspired a sense of partnership and trust. This factor may be particularly salient in rural communities where personal connections are valued as part of daily life.

Many HCPs may be unable to travel for quality CE and may be reluctant to participate in online training, but it appears cost-effective to partner with existing healthcare organizations and physician groups to maximize attendance. Little research is focused on marketing of CE programs, particularly in rural areas. This study addresses this gap and suggests formal advertisement is relatively less cost-effective. In times of limited financial resources for providing quality CE, cost-effective promotion of programs is crucial.
